# Multidisciplinary design optimisation of lattice-based battery housing for electric vehicles

**DOI:** 10.1038/s41598-024-60124-4

**Published:** 2024-05-28

**Authors:** Jier Wang, Maximilian Schutzeichel, Benedikt Plaumann, Thomas Kletschkowski, Ajit Panesar

**Affiliations:** 1 IDEA Lab, Department of Aeronautics, Imperial College London, London, SW7 2AZ UK; 2 Department of Automotive and Aeronautical Engineering, Hamburg University of Applied Sciences, 20099 Hamburg, Germany; 3 Airbus Operations GmbH, Kreetslag 10, 22021 Hamburg, Germany

**Keywords:** Lattice structures, Triply periodic minimal surfaces (TPMS), Battery housing, Topology optimisation (TO), Engineering, Energy infrastructure

## Abstract

Batteries with high energy densities become essential with the increased uptake of electric vehicles. Battery housing, a protective casing encapsulating the battery, must fulfil competing engineering requirements of high stiffness and effective thermal management whilst being lightweight. In this study, a graded lattice design framework is developed based on topology optimisation to effectively tackle the multidisciplinary objectives associated with battery housing. It leverages the triply periodic minimal surfaces lattices, aiming for high mechanical stiffness and efficient heat dissipation considering heat conduction and convection. The effectiveness of the proposed framework was demonstrated through the battery housing design, showcasing its ability to address multidisciplinary objectives as evidenced by the analysis of the Pareto front. This study identifies the potential of lattices in lightweight applications incorporating multiphysics and offers an efficient lattice design framework readily extended to other engineering challenges.

## Introduction

Electric vehicles (EVs) contribute to a reduction in emissions, promoting environmentally sustainable transportation. The global shift from traditional fossil-fuel-powered vehicles to EVs is underway, as evidenced by the UK government’s commitment to a zero-emission mandate for all new cars by 2035^1^, the EU’s plan to ban petrol and diesel cars by 2035^2^ and the widespread implementation of EV promotion policies across various regions. Along with the rising number of EVs, the battery, a crucial component of EVs, is evolving rapidly with increased energy density and battery life requirements^3^. The high energy density necessitates robust battery protection to prevent large deformation and fracture, potentially leading to thermal runaway, and requires efficient thermal management to avoid overheating, which may cause irreparable damage. Battery housing is a common solution for ensuring the integrity of the battery while maintaining a suitable operating temperature. Current battery housing designs^4,5^, typically made of solid metallic materials and located at the bottom of the vehicle, are usually heavy to ensure adequate protection. To progress the state-of-the-art battery housing design, efforts have been devoted towards lightweight, high mechanical performance, and efficient thermal management^6^. Shui et al. performed design optimisation targeting lightweight, high natural frequency and high stiffness on the battery housing by tuning six size parameters of a conventional solid metallic design, like wall thicknesses and bottom plate thicknesses^7^. A battery housing with a wedge-shaped runner for air cooling was proposed and optimised by changing the positions of the air inlet and outlet, the width and angle of the wedge runner, and the clearance between battery cells, resulting in enhanced temperature uniformity and decreased maximum temperature^8^. An integrated methodology for EV battery housing design was proposed by Li et al.^9^, involving optimisation of size parameters for the air cooling system. However, the majority of reported studies consider a conventional solid battery housing design, limiting the design exploration by a constrained number of size parameters. Dhoke and Dalavi^10^ reviewed several lightweight designs for battery housing and listed several future research directions, where the necessity of incorporating advanced optimisation algorithms like topology optimisation (TO) for lightweight design was particularly noted. Yeong et al.^11^ claimed to have carried out TO for the battery housing design; however, the optimisation was limited to the bolt mounting structure, yielding minor improvements and consequently leaving the entire battery housing domain unexplored which would have offered significant gains if optimised. Therefore, to advance EV development, there is an urgent need to explore the implementation of advanced optimisation techniques in optimising battery housing. Alternative to the traditional solid design, the inclusion of porosity in battery housing offers weight saving without compromising mechanical or thermal performance. The composite phase change material (PCM) made of paraffin and high porosity copper foam was adopted for the battery thermal management, demonstrating effective temperature control through double-sided liquid cooling^12^. Heavy metallic housing components were replaced by fibre-reinforced plastics outer shell and aluminium foam infiltrated with PCM to achieve weight saving^13^. However, the potential to leverage controlled porosity and tailored properties of porous structures for local strengthening and efficient material utilisation remains unexplored. Lattices allow more systematic control of porosities whilst offering outstanding performance in energy absorption, thermal insulation, sound attenuation, and vibration isolation^14–16^ applications and have therefore been proposed for a number of engineering usecases. Triply Periodic Minimal Surfaces (TPMS), a type of lattice composed of non-self-intersecting, periodic surfaces in three principal directions, serve as a suitable choice for battery housing^17^. The geometry, while complex, is self-supportive in nature, allowing relatively easy fabrication by additive manufacturing (AM)^18,19^. Furthermore, TPMS can be parametrised and easily integrated into optimisation to afford robust design exploration studies. Panesar et al.^20^ proposed different strategies to generate graded lattice structures from TO results and demonstrated the enhanced mechanical performance and robustness of graded lattices when compared to the uniform counterparts. Combining a predefined, parameterised lattice type with TO-based methods enables the generation of optimal designs with lower computational effort compared to full-scale TO^21^. For example, concurrent multi-scale TO, where the macro- and the micro-scales are optimised simultaneously during the optimisation process, can generate high-performance structures with optimised micro lattices^22,23^. Multi-scale TO targeting multiphysics objectives, specifically aimed at enhancing thermal conductivity and mechanical stiffness of lattice structures, has been undertaken by Ali and Shimoda^24^. However, existing multi-scale TO methods do not account for thermal convection, which limits their applicability in the design of EV battery housing. This paper proposes a first-of-a-kind TO-based framework to tackle the multidisciplinary design challenge associated with EV battery housing using multi-scale structures. From the perspective of multi-objective optimisation, a key contribution is the embedding of thermal convection characteristics, which enables the simultaneous consideration of mechanical vibration, thermal conduction and convection within the framework. Additionally, the optimised design adopts a novel multi-scale structure, where the grading of TPMS lattice results in improved performance in this combined thermo-mechanical case. The outline of the paper is as follows. Firstly, the overall battery housing design case is detailed. Secondly, the adopted TPMS lattice cell is introduced. Subsequently, the optimisation framework is presented, including the sensitivity calculation through adjoint analysis and the formulation of a multidisciplinary objective function. Finally, the lattice-based battery housing design solutions are reported and the effectiveness of the proposed framework is discussed.

## Methodology

### Problem definition: battery housing

Due to increased demand on energy density in EVs, battery cell geometries offering higher packing efficiency have gained traction. An example of this is the blade battery, which is typically utilised in a parallel configuration protected within a vehicle’s battery housing. Herein, we consider the battery housing enclosing blade batteries as a design case with the objectives of minimising its deformation under vibration and maximising heat dissipation during operation. The following subsections outline the overarching problem, detail the load cases and boundary conditions, specify the dimensions considered and state any modelling assumptions made.

#### Battery housing dimensions

**Figure 1:**
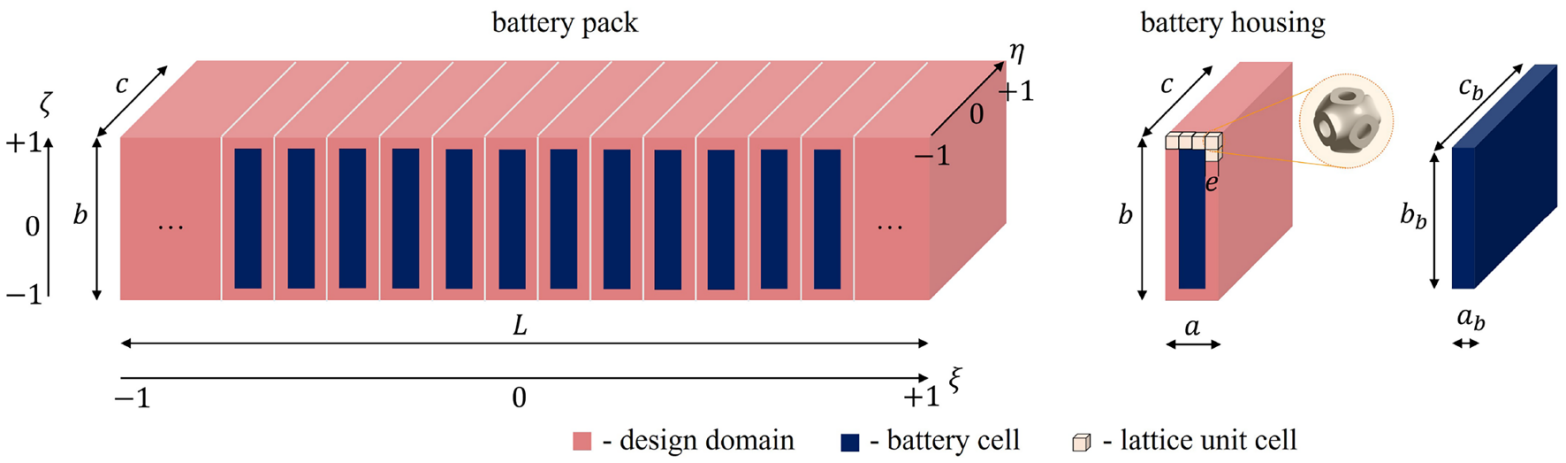
Illustrations of the battery pack, battery housing and battery cell.

Utilising the actual dimensions of the blade battery and accounting for real-world applications^25^, a battery pack model is created as illustrated in Fig. 1, with the dimensions summarised in Table 1 . A single battery housing unit is regarded as a representative component of the entire battery pack, and the dimensions are related to the overall size as entailed in Eq. (1). The design domain is intended to be occupied by lattices. Please note that some dimensions are subtly adjusted to ensure that they can be evenly divided by a chosen lattice unit cell edge length. Aluminium is chosen as the base material for lattice structures. The relevant properties of aluminium are detailed in Table 2.

**Table 1:** Dimensions for the battery pack, battery housing and battery cell.

Term	Symbol	Value	Unit
No. of battery cells in the battery pack	$N_b$	78	–
Dimension of battery pack along ξ axis	*L*	2106.0	mm
Dimension of battery housing along ξ axis	*a*	27.0	mm
Dimension of battery housing along η axis	*c*	1309.5	mm
Dimension of battery housing along ζ axis	*b*	135.0	mm
Dimension of battery cell along ξ axis	$a_b$	13.5	mm
Dimension of battery cell along η axis	$c_b$	1309.5	mm
Dimension of battery cell along ζ axis	$b_b$	121.5	mm
Edge of lattice unit cell	*e*	6.75	mm

**Table 2:** Table of material properties.

Term	Symbol	Value	Unit
Density	$\rho _0$	2700.0	$\text {kg} / \text {m}^3$
Young’s modulus	$E_0$	69.0	GPa
Poisson’s Ratio	$\mu _0$	0.33	
Thermal conductivity	$\kappa _0$	237.0	$\text {W}/(\text {mK})$

1 $$\begin{aligned} \begin{aligned} a_b&= 2 \cdot e \\ b_b&= 18 \cdot e \\ c_b&= 194 \cdot e \\ a&= a_b + 2 \cdot e \\ b&= b_b + 2 \cdot e \\ c&= 194 \cdot e \\ L&= N_b \cdot a \end{aligned} \end{aligned}$$

#### Mechanical loadcase

To maintain normal operation in EVs, the battery pack is required to function effectively under mechanical loads, particularly vibration loading. The lowest frequency is commonly considered as the most critical vibration condition, therefore the first mode of vibration, which manifests as a torsional deformation, is chosen as the mechanical loading scenario in this work. Modal analysis determines the eigenfrequencies and the mode shapes of a body. The mode shapes represent the deformation pattern which results once the body is excited by a harmonic sinusoidal force. In Finite Element (FE) formulation, the undamped eigenvalue problem is

2 $$\begin{aligned} ({{\textbf {K}}}-\omega ^2{{\textbf {M}}}){{\textbf {u}}} ={{\textbf {0}}}\text {,} \end{aligned}$$

where **K** is the stiffness tensor, $\omega$ is the angular frequency, **M** is the mass tensor and **u** is the eigenmode displacement vector.

To extract an exemplary deformation pattern serving as a representative load case, a cuboid volume of 2106.0 mm $\times$ 1309.5 mm $\times$ 135.0 mm is created, and modal analysis is conducted in COMSOL Multiphysics^26^ which considers the eigenvalue problem in Eq. (2).

**Figure 2:**
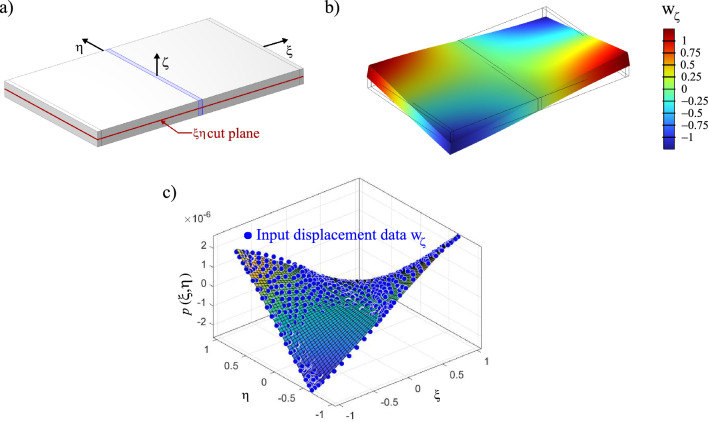
(**a**) Cuboid volume for modal analysis, (**b**) visualised displacement result related to the first mode of vibration at $\omega _\text {1}$ of a normalised modal load, (**c**) resulting displacement pattern on $\xi -\eta$ cut surface and resulting polynomial $p(\xi ,\eta )$.

The cuboid, which represents the battery pack, is assumed to be isotropic and made from homogeneous aluminium. The outer dimensions are informed by the chosen battery pack as entailed in Table 1. In addition, the authors chose to simplify the mechanical case. Accordingly, no specific vehicle system setup is considered. However, to prevent rigid body motions in the model while not influencing the mode shapes, a spring foundation^27^ on the lower surface $\Gamma _\zeta ^{-}$ is defined (in the coordinate system shown in Fig. 2a) as

3 $$\begin{aligned} {{\textbf {f}}}_s=k_s{{\textbf {u}}}(\Gamma _\zeta ^{-}) \text {,} \end{aligned}$$

where ${{\textbf {f}}}_s$ is a force per unit area field, $k_s$ is the spring constant and ${{\textbf {u}}}(\Gamma _\zeta ^{-})$ is the deformation of the lower surface of the plate. Since this spring foundation represents a stiffness boundary condition, the spring stiffness $k_s$ contributes to the overall stiffness matrix **K** of the eigenvalue problem. A low spring constant of $k_s=1 \text {N}/\text {m}^3$ is chosen. Compared to the overall stiffness of the component made from aluminium with significant cross sections, the effect of this spring foundation definition on the eigenfrequencies $\omega _\text {i}$ and eigenmodes ${{\textbf {u}}}_\text {i}$ is considered to be negligible. Furthermore, the volume is discretised with linear tetrahedral finite elements. Solving the eigenvalue problem in COMSOL Multiphysics provides the set of eigenfrequencies $\omega _\text {i}$ along with the related eigenmode displacement vectors ${{\textbf {u}}}_\text {i}$.

The first eigenfrequency is found at $\omega _\text {1}=155.8 \text { Hz}$ and the related deformation pattern is analysed related to the cuboid. To identify the equivalent boundary conditions for this battery pack, the cuboid is cut by the $\xi -\eta$ base plane (($\zeta =0$), see Fig. 2a). This position is considered to properly represent the displacement field ${\textbf{w}}$ for the first mode of vibration. As this deformation pattern occurs at the first eigenfrequency, this mechanical condition is considered to be the most severe with respect to absolute deformation magnitude. In Fig. 2b the amplified deformation pattern is visualised. To make the displacement field applicable as a boundary condition for the optimisation procedure, the $\zeta$ component of the displacement field $\text {w}_\zeta$ is extracted for each FE node on the $\xi -\eta$ cut plane (Fig. 2a). From this discrete displacement field $\text {w}_\zeta (\xi ,\eta )$ a surface interpolation polynomial of the form

4 $$\begin{aligned} p(\xi ,\eta ) =p_{00} + p_{10} \cdot \xi +p_{01} \cdot \eta + p_{20} \cdot \xi ^2 +p_{11} \cdot \xi \cdot \eta +p_{02} \cdot \eta ^2 \end{aligned}$$

is calculated in Matlab^28^. The resulting polynomial is visualised as a surface function in Fig. 2c alongside the computed displacement field of $\text {w}_\zeta (\xi ,\eta )$. The fitness measure of this polynomial was $R^2$ > 0.95. Table 3 reports the coefficients of this polynomial.

**Table 3:** Table of polynomial coefficients obtained from results for $w_\zeta$.

Symbol	$p_{00}$	$p_{10}$	$p_{01}$	$p_{20}$	$p_{11}$	$p_{02}$
Value	$9.93\text {e}^{-10}$	$9.49\text {e}^{-10}$	$-\,9.76\text {e}^{-11}$	$-\,1.55\text {e}^{-9}$	$-\,2.66\text {e}^{-6}$	$4.46\text {e}^{-10}$

**Figure 3:**
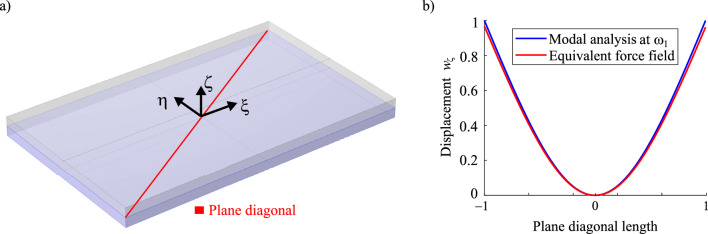
(**a**) Cuboid volume with plane diagonal visualised, (**b**) displacement results along base plane diagonal from modal analysis and equivalent force field boundary conditions.

The polynomial is then adopted to serve as the function of an equivalent distributed force for both the upper $\Gamma _\zeta ^{+}$ and lower $\Gamma _\zeta ^{-}$ surfaces of the cuboid volume. Therefore, the polynomial reads

5 $$\begin{aligned} F(\xi ,\eta ) =F_0 \cdot (p_{00} + p_{10} \cdot \xi +p_{01} \cdot \eta + p_{20} \cdot \xi ^2 +p_{11} \cdot \xi \cdot \eta +p_{02} \cdot \eta ^2) \end{aligned}$$

where $F_0$ represents a tuning factor for the force field magnitude. This parameter is set to $F_0=130000 {\text {kN}}/{\text {m}^2}$, which is defined by analysing the resulting displacement field of the cuboid volume from a static load case. The resulting displacements in $\zeta$ direction under the equivalent force are computed and compared with the displacement data $w_\zeta$ of modal analysis along the diagonal line of the $\xi -\eta$ cut plane (see Fig. 3). Both curves are in good agreement with a fitness measure of $R^2$ > 0.95. As the equivalent distributed force $F(\xi ,\eta )$ mimics the actual dynamic deformation pattern given by modal analysis, it is considered as the load case for the mechanical problem in our structural optimisation.

#### Thermal loading

Effectively managing the thermal conditions of batteries is a crucial aspect of the advancement of EVs. This work focuses on steady-state heat dissipation of battery cells operating at maximum power, mimicking the critical thermal state during fast charging scenarios. To prevent the battery operating temperature from surpassing the acceptable range, it is imperative to incorporate thermal considerations into the design of the battery housing.

Recall from Fig. 1 wherein the battery pack is introduced, we consider heat generation from the battery cells to be uniformly distributed on the battery cell surface, excluding the surfaces at $\eta =-1$ and $\eta =+1$. Both heat conductivity and heat convection are taken into account. For heat convection, air cooling is chosen, providing the opportunity to utilise incoming air when the EV is in motion. However, it should be noted that the peak heat generated by the battery typically occurs during charging when the EV is stationary. Therefore, active air cooling is considered with the direction of airflow set along the $\eta$ axis, aligned with the longest dimension of the battery cell. To involve thermal convection within the design optimisation, the temperature field of airflow is required as the boundary condition. This is computed using a simplified one-dimensional steady-state fluid analysis for a single battery housing (refer to Fig. 1) with the relevant parameters listed in Table 4. The initial airflow temperature, which is associated with the ambient temperature, is assumed to be 280 K herein. For simplicity, the lattice-relevant properties and parameters are calculated using a relative density of $\rho =0.2$. The heat input from the battery cells is assumed to be uniformly distributed along the $\eta$ axis.

**Table 4:** Parameters in fluid analysis.

Term	Symbol	Value	Unit
Heat dissipated per second per battery cell	${\dot{q}}$	200	$\text {W}$
Convection coefficient (forced air convection)^29^	$h_f$	32	$\text {W}/(\text {m}^2 \text {K})$
Speed of the airflow	$v_f$	5	$\text {m}/\text {s}$
Specific heat capacity of air	$c_p$	1218	$\text {J}/(\text {m}^3 \text {K})$
Initial airflow temperature	$T_{f0}$	280	$\text {K}$

The airflow channel refers to the battery housing cross-sectional area in the $\xi -\zeta$ plane. The cross-sectional area of the airflow channel ($A_f$) is calculated as

6 $$\begin{aligned} A_f = a\cdot b - a_b\cdot b_b = 44 e^2 \end{aligned}$$

Based on Eq. (20), when $\rho =0.2$, the conductivity of SP lattice is

7 $$\begin{aligned} \kappa _l = 0.1334 \kappa _0 \end{aligned}$$

According to Eq. (21), the surface area for a single SP lattice cell is

8 $$\begin{aligned} S = 4.62 e^2 \end{aligned}$$

Perpendicular to the airflow direction ($\eta$ axis), there are 44 lattice cells on the $\xi -\zeta$ plane in a battery housing, therefore, the lattice surface area for heat convection per unit length can be calculated as

9 $$\begin{aligned} A_h = 44 \cdot S / e = 203.28 e \end{aligned}$$

The above-calculated parameters are summarised in Table 5 and used in the following fluid analysis.

**Table 5:** Additional parameters in fluid analysis.

Term	Symbol	Value*	Unit
Cross-sectional area of the airflow channel	$A_f$	$4.62 e^2$	$\text {m}^2$
Heat convection surface area per meter along the airflow channel	$A_h$	203.28*e*	$\text {m}$
Heat conductivity of lattice	$\kappa _l$	$0.1334 \kappa _0$	$\text {W}/(\text {m} \text {K})$

Considering the temperature fields of the lattice-based battery housing and airflow are functions of physical coordinate *y* ($y=\frac{c}{2} \cdot \eta$), and are represented using $T_l(y)$ and $T_f(y)$, respectively. The temperature change of airflow along the *y* axis is equal to the heat transfer through convection:

10 $$\begin{aligned} h_f A_h (T_l - T_f) = v_f A_f c_p \frac{dT_f}{dy} \end{aligned}$$

In the lattices, the heat generated by the battery cells is transferred through conduction and convection, reaching a steady state:

11 $$\begin{aligned} \frac{{\dot{q}}}{c} = h_f A_h (T_l - T_f) - \kappa _l \frac{d^2 T_l}{d y^2} A_f \end{aligned}$$

The assumption has been made that there is no heat conduction at the tips of the battery housing:

12 $$\begin{aligned} \begin{aligned}{}&\frac{dT_l}{dy} _{y=-\frac{c}{2}} = 0 \\&\frac{dT_l}{dy} _{y=\frac{c}{2}} = 0 \end{aligned} \end{aligned}$$

where $y=-c/2$ and $y=c/2$ refer to the airflow inlet and outlet, respectively.

Additionally, the temperature of the airflow at the channel’s inlet is

13 $$\begin{aligned} T_f (-\frac{c}{2}) = T_{f0} \end{aligned}$$

**Figure 4:**
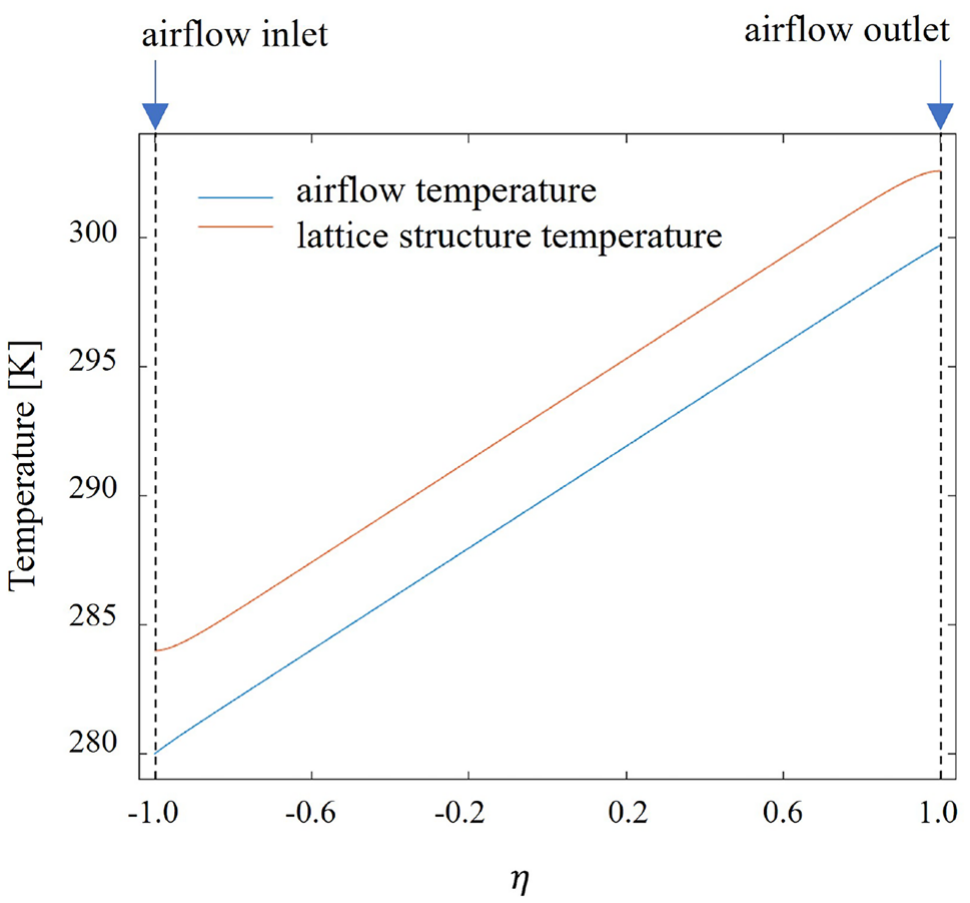
Temperature fields of airflow and lattice structure.

The solution is plotted with $\eta$ as the X axis in Fig. 4, where $\eta =-\,1.0$ and $\eta =1.0$ indicate the airflow inlet and outlet, respectively. The figure displays temperatures for both the airflow and the lattice structure, with the airflow temperature closely adhering to a linear function of $\eta$, while the temperature of the lattice structure consistently remains approximately 4 K higher than the airflow. An approximated linear function for the airflow temperature field is captured:

14 $$\begin{aligned} T_f (\eta ) = 289.85+9.85 \eta \end{aligned}$$

The lattice structure’s temperature is increased by approximately 20 K, with the highest temperature reaching 25 K above the ambient temperature. It’s important to note that the absolute temperature value depends on the ambient temperature, which is the airflow temperature at the inlet. However, the temperature increase is evidently constrained, keeping the battery operating temperature under 323 K. Exceeding this temperature threshold may lower the charging efficiency or impair battery longevity^30^.

### Lattice cell topology and property

#### Lattice cell generation

**Figure 5:**
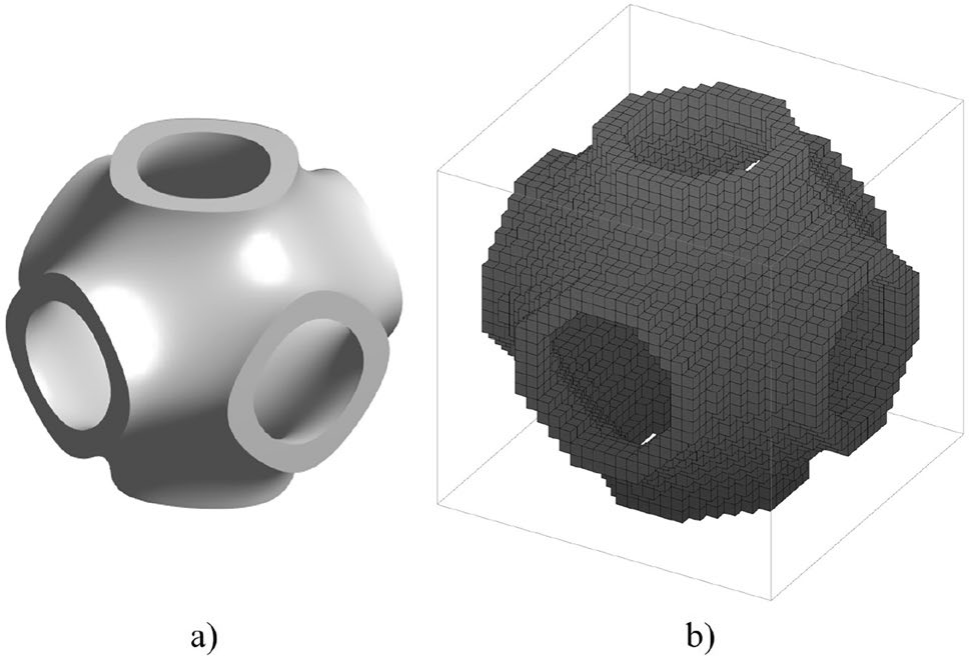
(**a**) SP lattice and (**b**) discretised mesh for SP lattice.

TPMS lattice is created using level-set equations. For example, the Schwarz primitive (SP) (as shown in Fig. 5a) is generated through

15 $$\begin{aligned} \left| cos(\omega x) + cos(\omega y) + cos(\omega z) \right| \leqslant t \end{aligned}$$

where the isovalue *t* serves as a control parameter for the offset from the zero level-set, and $\omega$ is the TPMS function periodicity with the value $\omega =2 \pi / e$ for lattice cell edge length of *e*.

For SP, the value of *t* governs the bounds for the solid phase. As the parameter *t* is modified, the cell volume fraction ($V_f$) - or, alternatively, the cell relative density ($\rho$) - adjusts accordingly. SP is selected as an example to illustrate the proposed lattice design framework, with lattice relative density $\rho$ as the design variable. Bounds on the relative density $\rho$ are imposed (eqn. below) to ensure minimum wall thickness for printability and allow for convective transport to occur.

16 $$\begin{aligned} 0.1 \le \rho \le 0.5 \end{aligned}$$

#### Homogenisation method: calculation of cell property

To obtain the effective properties of lattice cells, homogenisation method is employed to calculate both the homogenised mechanical constitutive matrix (${\textbf{D}}_H$) and the homogenised thermal conductivity ($\mathbf {\kappa }_H$).

The constitutive relation in three-dimensions is

17 $$\begin{aligned} \mathbf {\sigma } = {\textbf{D}} \mathbf {\varepsilon } \end{aligned}$$

where $\mathbf {\sigma }=\left[ \sigma _{11}, \sigma _{22}, \sigma _{33}, \sigma _{23}, \sigma _{31}, \sigma _{12}\right] ^T$ and $\mathbf {\varepsilon }=\left[ \varepsilon _{11}, \varepsilon _{22}, \varepsilon _{33}, \varepsilon _{23}, \varepsilon _{31}, \varepsilon _{12}\right] ^T$ are the stress tensor and strain tensor, respectively.

Considering the symmetry of SP, the homogenised mechanical constitutive matrix can be represented using three independent parameters $D_{1111}, D_{1122}, D_{2323}$:

18 $$\begin{aligned} {\textbf{D}}_H = \left[ \begin{matrix} D_{1111}&{} D_{1122}&{} D_{1122}&{} 0&{} 0&{} 0&{} \\ D_{1122}&{} D_{1111}&{} D_{1122}&{} 0&{} 0&{} 0&{} \\ D_{1122}&{} D_{1122}&{} D_{1111}&{} 0&{} 0&{} 0&{} \\ 0&{} 0&{} 0&{} D_{2323}&{} 0&{} 0&{} \\ 0&{} 0&{} 0&{} 0&{} D_{2323}&{} 0&{} \\ 0&{} 0&{} 0&{} 0&{} 0&{} D_{2323}&{} \end{matrix} \right] \end{aligned}$$

The SP unit cell is discretised into a voxel-based structure with the resolution $30\times 30\times 30$ (as shown in Fig. 5b) and then run through homogenisation method^31^ to obtain the homogenised properties ${\textbf{D}}_H$ and $\mathbf {\kappa }_H$.

#### Development of surrogate models for lattice properties

**Figure 6:**
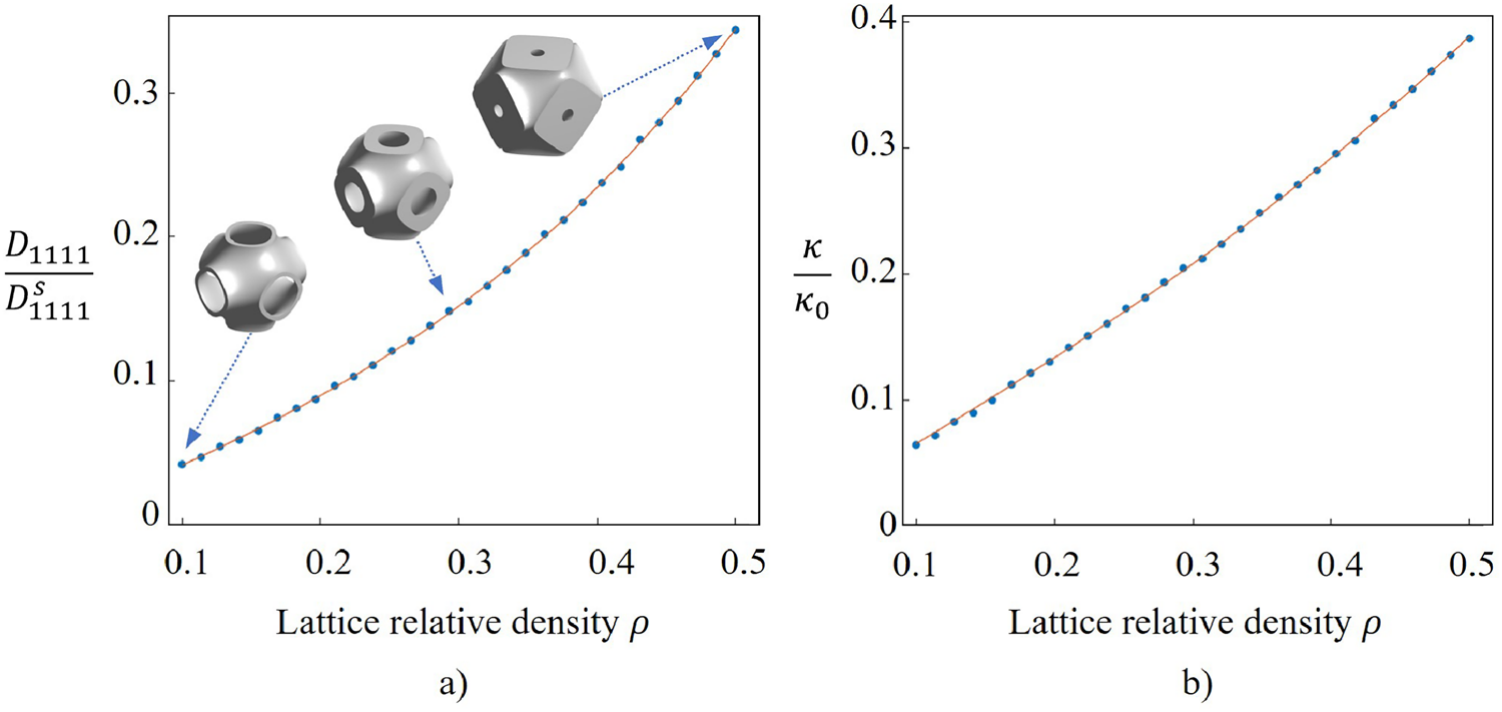
Regression models for the relationships between cell properties (**a**) ${D_{1111}} /{D^{s}_{1111}}$ (**b**) ${\kappa } /{\kappa _{0}}$) and relative density $\rho$.

In order to perform structural optimisation over the cell relative density ($\rho$), relationships between lattice cell properties and variable $\rho$ await figuring out. Cell properties include the surface area (*S*) of lattice unit cell, the mechanical constitutive matrix (${\textbf{D}}_H$) and the thermal conductivity ($\mathbf {\kappa }_H$). Polynomial regression using the method of least squares is adopted to model the relationships. 30 points are sampled uniformly within the lower and upper bounds of $\rho$ as data points. Regression results are shown in Fig. 6.

To summarise, the regression models for the three independent parameters of the constitutive matrix:

19 $$\begin{aligned} \begin{aligned}{}&\frac{D_{1111}}{D^{s}_{1111}} = 1.0700 \cdot \rho ^3 + 0.4059 \cdot \rho \\&\frac{D_{1122}}{D^{s}_{1122}} = 0.1734 \cdot \rho ^3 + 0.2503 \cdot \rho \\&\frac{D_{2323}}{D^{s}_{2323}} = 0.2689 \cdot \rho ^3 + 0.1924 \cdot \rho \end{aligned} \end{aligned}$$

where the superscript ’*s*’ refers to the bulk solid material.

Similarly, the thermal conductivity has the following expression

20 $$\begin{aligned} \frac{\kappa }{\kappa _{0}} = 0.4617 \cdot \rho ^3 + 0.6487 \cdot \rho \end{aligned}$$

A quadratic fit was deemed adequate for the surface area:

21 $$\begin{aligned} \frac{S}{e^2} = -3.6988 \cdot \rho ^2 + 0.4954 \cdot \rho + 4.666 \end{aligned}$$

### Multidisciplinary lattice design framework

#### Optimisation formulation

In this multidisciplinary design case associated with structural stiffness and heat dissipation, the optimisation problem is defined as Eq. (22), with details presented in the following paragraphs.

22 $$\begin{aligned} \begin{aligned} \min _{\rho } \ {}&C = w_m \cdot \frac{C_m-C_m^{min}}{C_m^{max}-C_m^{min}} + w_t \cdot \frac{C_t-C_t^{min}}{C_t^{max}-C_t^{min}} \\ s.t. \ {}&w_m + w_t =1 \\&K_m U = F \\&K_c T + K_v (T - T_f )= Q \\&g(\rho ) \le 0 \\&\rho _e \in \left[ 0.1,0.5 \right] , \forall e \end{aligned} \end{aligned}$$

where *C* is the compliance, which is the objective of the optimisation problem. *w* is the weight assigned to the competing objectives. Subscripts *m* and *t* denote mechanical and thermal, respectively. Superscripts ’*min*’ and ’*max*’ refer to the upper and lower bounds of the objective. In the mechanical equilibrium equation (i.e. $K_m U = F$), $K_m$, *U*, and *F* are the stiffness matrix, displacement vector, and force vector. In the thermal equilibrium equation (i.e. $K_c T + K_v (T - T_f )= Q$), $K_c$, $K_v$, *T*, $T_f$, and *Q* are the heat conductivity matrix, heat convection matrix, structural temperature vector, cooling airflow temperature vector, and thermal load vector, respectively. The function *g* represents the total volume constraint. $\rho$ is the vector of lattice unit cell relative densities in the design domain and serves as the design variable of the optimisation problem. The subscript *e* indicates an element (also a lattice unit cell). The structural compliance $C_m$ and the thermal compliance $C_t$ are defined as Eq. (23). FE analysis is adopted to assess the mechanical compliance and thermal compliance of the lattice structure, with each FE element representing the homogenised property of a lattice unit cell. The whole design domain is meshed using eight-node hexahedral elements and analysed using full integration.

23 $$\begin{aligned} \begin{aligned}{}&C_m = F^T U \\&C_t = Q^T T \end{aligned} \end{aligned}$$

As presented in Eq. (22), the weighted sum objective function is formulated by incorporating coefficients to assess the trade-offs between the multiple objectives, and normalising the objectives considering the disparities in objective magnitudes and maximum objective improvements. The global programming method is employed herein for its robustness^32,33^. The ‘*min*’ value is calculated through optimising the single objective, whilst the ’*max*’ value is determined by optimising the ’other’ objective. For example, $C_m^{min}$ and $C_t^{max}$ are the performances of the design optimised for the single mechanical objective. The thermal equilibrium equation herein involves thermal conductivity and thermal convection. For simplicity, $K_A$ is defined as

24 $$\begin{aligned} K_A = K_c+K_h \end{aligned}$$

and the thermal equilibrium equation is modified to

25 $$\begin{aligned} K_A T - K_v T_f = Q \end{aligned}$$

A constraint is imposed on the design variable ($\rho _e$) as mentioned in Lattice cell topology and property section.

26 $$\begin{aligned} 0.1 \le \rho _e \le 0.5 \end{aligned}$$

Besides the constraint on each design variable, a constraint $g(\rho )$ is imposed on the global volume as in standard TO.

27 $$\begin{aligned} g(\rho ) = \sum _{e}^{} \rho _e \upsilon _e - V_f^* \sum _{e}^{} \upsilon _e \le 0 \end{aligned}$$

where $V_f^*$ is the target volume fraction of the design domain; $\upsilon _e$ is the element volume, a constant value that is related to the edge length of the element:

28 $$\begin{aligned} \upsilon _e = e^3 \end{aligned}$$

#### Sensitivity calculation with adjoint analysis

For solving the optimisation problem defined by Eq. (22) using the gradient-based solver, the sensitivity of the weighted objective *C* over design variables $\rho _e$ is needed, which is elaborated in the following paragraphs, employing the adjoint method. From a modified expression of *C*:

29 $$\begin{aligned} C = \frac{w_m}{C_m^{max}-C_m^{min}} \cdot (F^T U-C_m^{min})+ \frac{w_t}{C_t^{max}-C_t^{min}} \cdot (Q^T T-C_t^{min}) \end{aligned}$$

a new adjoint parameter is created:

30 $$\begin{aligned} {\widehat{C}} = C + \frac{w_m}{C_m^{max}-C_m^{min}} \cdot \lambda _t ^T ( K_mU-F )+ \frac{w_t}{C_t^{max}-C_t^{min}} \cdot \lambda _t ^T ( K_A T - K_vT_f-Q ) \end{aligned}$$

The sensitivity of the new adjoint parameter ${\widehat{C}}$ with respect to the design variables has the form

31 $$\begin{aligned} {{\widehat{C}}}' = \frac{w_m}{C_m^{max}-C_m^{min}} \cdot ( \frac{\partial \Phi }{\partial U} {U}' + \lambda ^T (K_m'U+K_mU') ) + \frac{w_t}{C_t^{max}-C_t^{min}} \cdot ( \frac{\partial \Phi }{\partial T} {T}' + \lambda _t^T (K_A'T+K_AT'-K_v'T_f-K_vT_f') ) \end{aligned}$$

where ${'}$ is to indicate the derivative over design variable ${\rho _e}$. $\lambda _m$ and $\lambda _t$ are the newly introduced adjoint state variables, for which the details can be found from adjoint analysis in TO^34^.

An assumption is made here to regard the change of $T_f$ over ${\rho _e}$ as negligible:

32 $$\begin{aligned} T_f' = 0 \end{aligned}$$

By collecting terms for $U'$ and $T'$, and setting them as 0, the expressions for $\lambda _m$ and $\lambda _t$ are derived:

33 $$\begin{aligned} \begin{aligned} \lambda _m^T&= - U \\ \lambda _t^T&= - Q^T K_A^{-1} \end{aligned} \end{aligned}$$

Replacing $T_f'$, $\lambda _m$ and $\lambda _t$ in Eq. (31), it leads to

34 $$\begin{aligned} {{\widehat{C}}}' = \frac{w_m}{C_m^{max}-C_m^{min}} \cdot ( -U^T K_m' U ) + \frac{w_t}{C_t^{max}-C_t^{min}} \cdot ( - Q^T K_A^{-1} (K_A'T-K_v'T_f) ) \end{aligned}$$

The sensitivities $K_m'$, $K_A'$ and $K_v'$ in Eq. (34) can be calculated through the chain rule

35 $$\begin{aligned} \begin{aligned} {K_m}'&= \frac{\partial K_m }{\partial D_j} \frac{\partial D_j }{\partial \rho _e} \ (j=1111,1122,2323) \\ {K_A}'&= \frac{\partial (K_c+K_v) }{\partial \rho _e} = \frac{\partial K_c }{\partial \kappa } \frac{\partial \kappa }{\partial \rho _e} + \frac{\partial K_v }{\partial S} \frac{\partial S }{\partial \rho _e} \\ {K_v}'&= \frac{\partial K_v }{\partial S} \frac{\partial S }{\partial \rho _e} \end{aligned} \end{aligned}$$

with ${\partial D_j} / {\partial \rho _e}$, ${\partial \kappa } / {\partial \rho _e}$ and ${\partial S} / {\partial \rho _e}$ directly derived from the surrogate models in Lattice cell topology and property section, ${\partial K_m }/{\partial D_j}$, ${\partial K_c }/{\partial \kappa }$, and ${\partial K_v }/{\partial S}$ attained according to element stiffness matrix formula and the global stiffness matrix assembly rule.

## Results and discussion

The effectiveness of the proposed multidisciplinary design framework is demonstrated through the generation of a Pareto front for the battery housing design, with weight pairs for mechanical and thermal objectives listed in Table 6. The weights are chosen ranging from 0.00 to 1.00 in increments of 0.25 to create a Pareto front, allowing the solution that considers a 50/50 mechanical and thermal objective to be included. Herein, a target volume fraction $V_f^*$ of 30% is used. $C_m^{min}$ and $C_t^{max}$ are obtained by evaluating the optimised designs from the single mechanical objective optimisation (case I), while $C_m^{max}$ and $C_t^{min}$ are from the thermal optimisation (case V). A uniform lattice design is also evaluated for benchmarking purposes.

**Table 6:** Weights of different optimisation cases.

Case number	I	II	III	IV	V
$w_m$	1.00	0.75	0.50	0.25	0.00
$w_t$	0.00	0.25	0.50	0.75	1.00

**Figure 7:**
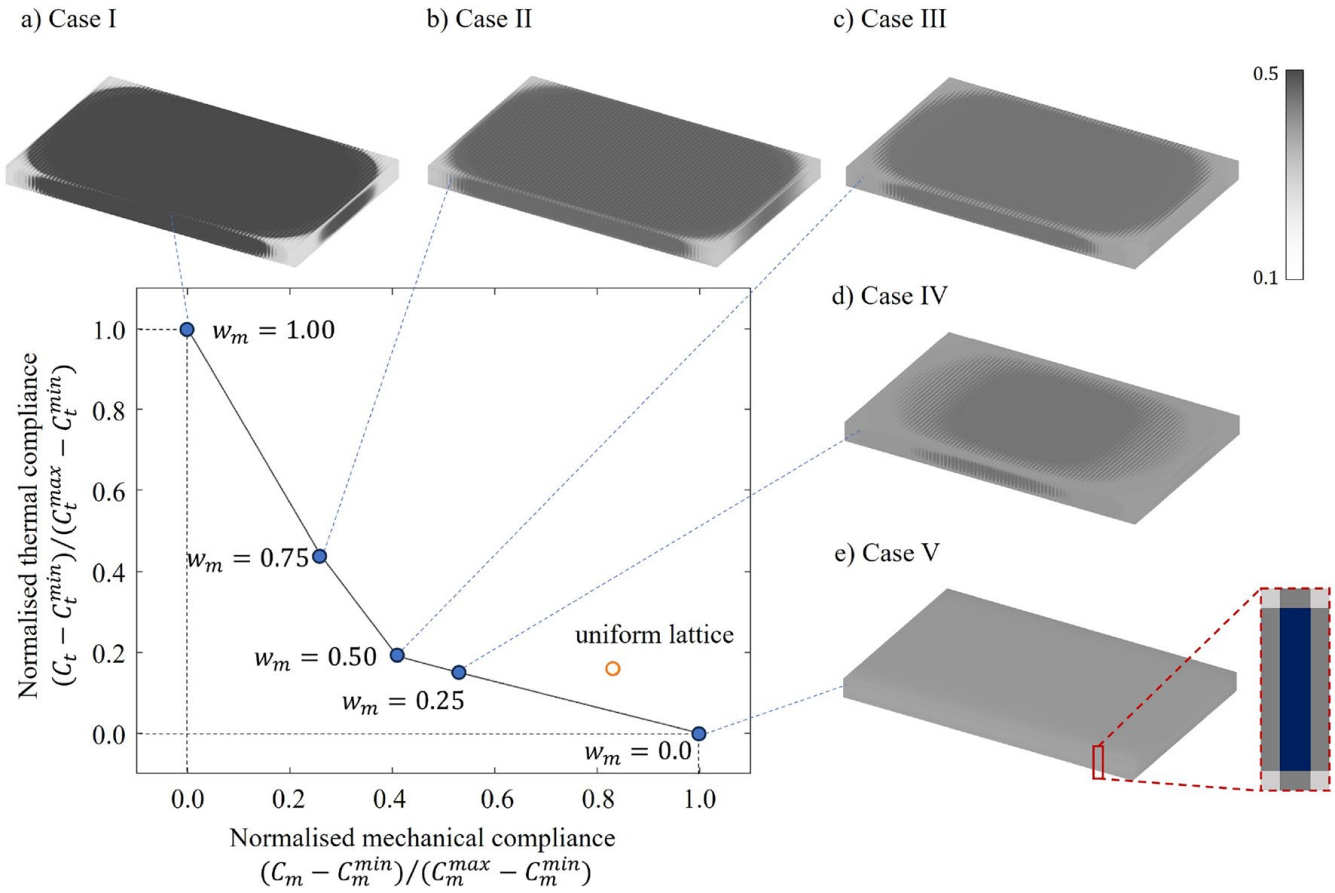
Pareto front and optimised lattice-based battery housing designs.

Figure 7 displays the optimised battery housing designs for the five cases, with the corresponding performances summarised in Table 7. From case I to case V, the weight assigned to the thermal objective gradually increases, leading to the change of optimised lattice structures from Fig. 7a–e. The improved thermal performance of the optimised lattice structure comes at the cost of decreased mechanical stiffness.

**Table 7:** Performance of the battery housing designs.

Weights*	$C_m (\times 10^{-5})$	$C_t(\times 10^{6})$	$\frac{C_m-C_m^{min}}{C_m^{max}-C_m^{min}}$	$\frac{C_t-C_t^{min}}{C_t^{max}-C_t^{min}}$
$w_m=1.00$	5.327	4.5940	0.00	1.00
$w_m=0.75$	6.535	4.5927	0.26	0.44
$w_m=0.50$	7.216	4.5921	0.41	0.19
$w_m=0.25$	7.798	4.5920	0.53	0.15
$w_m=0.00$	9.652	4.5917	1.00	0.00
Uniform lattice	9.167	4.5921	0.83	0.16

* $$w_t=1-w_m$$.

Viewed from the top ($\zeta =1$), the lattices in Fig. 7a tend to have higher $\rho$ in the centre ($\xi =0$, $\eta =0$) and lower $\rho$ at the corners ($\xi =\pm 1$, $\eta =\pm 1$), resembling an elliptical shape. This elliptic characteristic is also witnessed in all other cases considering mechanical loading. To understand this, one could consider a quarter of the battery pack (e.g. $\xi >0$ and $\eta <0$ ), where the loading can be assumed to be bending. Due to the antisymmetric case of the torsional loading, the centre is fixed, making the element close to it regarded as the root. From Euler beam theory, the curvature is inversely related to the second moment of cross-sectional area (*I*). Additionally, tip displacement can be understood to be influenced by its distance from the root and the rotations at the root, which is related to the curvature. As more material accumulates at the centre ($\xi =0,\eta =0$), a greater *I* is achieved in the middle of the battery pack, leading to smaller curvature at the root. Consequently, resulting in a lower displacement at the tips ($\xi =\pm 1,\eta =\pm 1$). Therefore, one can see how higher $\rho$ in the middle of the battery pack produces a stiffer structure.

**Figure 8:**
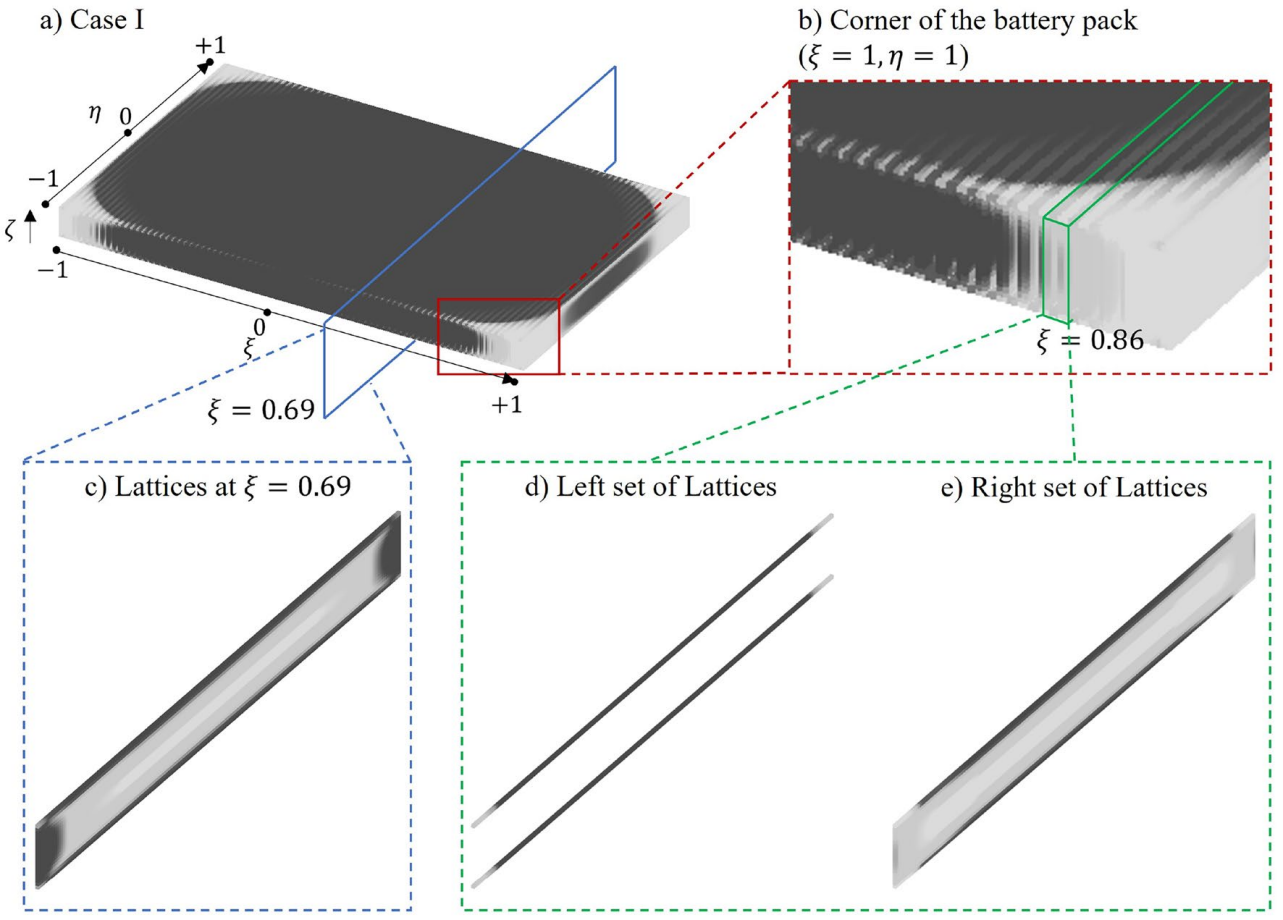
Relative density distribution for case I.

Figure 8 presents the results for case I, demonstrating a 42% reduction in mechanical compliance, which translates to a performance improvement of $1.7\times$ of that from the benchmark case.. From Fig. 8b (a zoomed-in plot close to line $\xi -1=\eta +1=0$), bands of lattices with higher $\rho$ can be observed on the top plane ($\zeta =1$). Two neighbouring sets of lattices located around $\xi =0.86$ are displayed as Fig. 8d,e, where (d) shows the left set of lattice cells that have a void region for $-0.9\leqslant \zeta \leqslant 0.9$ reserving for battery cell and (e) is the right set having lattices for $-1\leqslant \zeta \leqslant 1$. A comparison between (d) and (e) is summarised in Table 8, where (d) has total mass reaches 1/4 of (e) with only 9% lattice cell numbers. Additionally, (d) has $\rho _{avg}$ very close to the upper bound of $\rho$ and most lattices bounded by the upper limit of $\rho$, which indicates that having a thicker surface on the top and bottom of the battery pack could be considered to further improve the mechanical stiffness.

**Table 8:** Comparison between neighbouring $\eta -\zeta$ planes.

	Left set of lattices (shown in Fig. 8d)	Right set of lattices (shown in Fig. 8e)
Average $\rho$ ($\rho _{avg}$)	0.46	0.18
No. of lattice cells	388	4268
Total mass ($\times e^3$)	178	768

Figure 8c shows the $\eta -\zeta$ plane at $\xi =0.89$, where the four edges have a higher $\rho$ value. This can be explained considering the torsional rigidity, which is defined as *GJ*, with *G* being the shear module and *J* representing the second moment of area:

36 $$\begin{aligned} J = \int \int _{A}^{} r^2 dA \end{aligned}$$

where *A* is the area and *r* is the polar distance to the infinitesimal area element *dA*. Hence, more material at the edges with a greater distance from the centre line ($\eta =\zeta =0$) can increase the torsional rigidity and resist deformation under torsional vibration.

The advantage of multi-objective optimisation is demonstrated in cases II, III, and IV, where both mechanical and thermal objectives are considered. These cases maintain an elliptical characteristic that significantly enhances structural stiffness while strategically allocating more material to the corners for thermal management. As depicted in Fig. 7 and evaluated by normalised compliances, case III makes a minor trade-off in thermal performance but offers a mechanical improvement $\sim 2\times$ better than the uniform lattice. Case IV, while showing a modest improvement in thermal performance over the uniform lattice, marks a notable gain in mechanical stiffness, underscoring the effectiveness of multi-objective optimisation.

**Figure 9:**
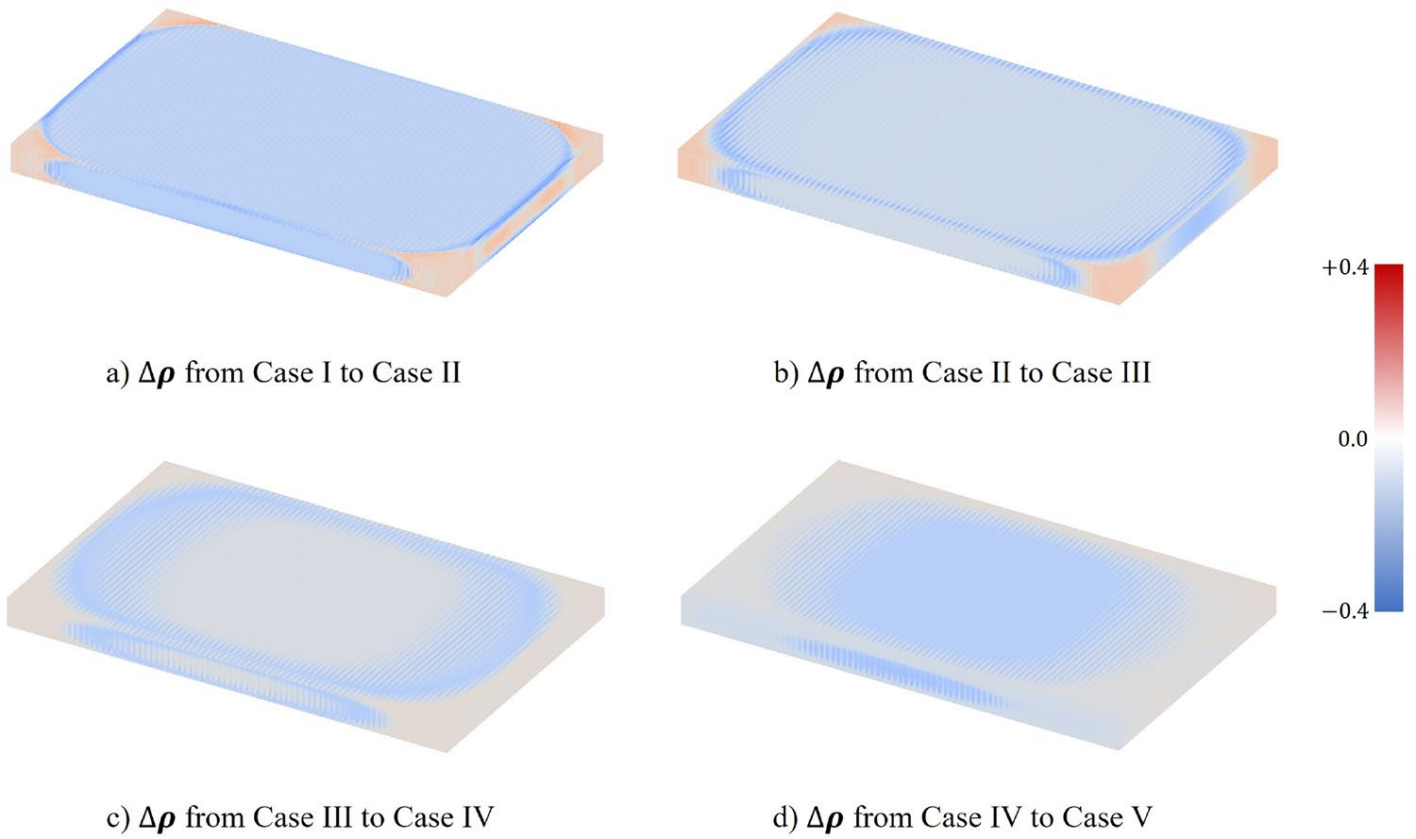
Variations of $\rho$ between cases.

To understand how the material allocation changes when the weights are altered in the multi-objective formulation, variations in the $\rho$ field ($\Delta \rho$) between neighbouring cases are plotted in Fig. 9. Positive values indicate an increase in $\rho$, whilst negative indicates a reduction. Considering the upper and lower bounds of $\rho$, the range of $\Delta \rho$ is within $-0.4 \leqslant \Delta \rho \leqslant 0.4$. From case I to II (Fig. 9a), the elliptic pattern fades slightly and large decreasements of $\rho$ only seen at the edges of the battery pack. From case II to IV (Fig. 9b,c), the elliptic pattern shrinks very slightly at the perimeter, as evidenced by the dominant grey region (i.e. neglectable change). Moving from case IV to V (Fig. 9d), more substantial changes in the elliptic pattern can be witnessed. With a higher weighting assigned to the thermal objective, the material reallocated from the middle to the corners, producing a more uniform lattice design. It can be therefore inferred that the optimisation is enforcing this elliptic feature to minimise the mechanical objective.

In the mono-functional case of thermal objective (case V), there’s a 6% improvement in normalised thermal compliance compared to the uniform lattice. As shown in Fig. 7e, the resulting design is close to the uniform lattice structure, as the thermal loads and boundary conditions applied are constant along $\eta$ axis, and a series of the same battery cells are stacked in $\xi$ axis. The zoomed-in section of Fig. 7e illustrates the repetitive pattern for each battery cell viewed from the front ($\eta =-1$). Small differences among elements can be observed in this zoomed-in section, where the elements that are face-connected to the battery cell have lattices with higher $\rho$ whilst the edge-connected ones have a lower $\rho$ value. Please recall, from Problem definition: battery housing section, we identified the correlation between the relative density and thermal conductivity and convectivity. A lower $\rho$ favours conduction whilst a higher $\rho$ promotes thermal convection (based on Eqs. 20 and 21). The face-connected elements have a larger number of nodes under thermal heat flux than the face-connected ones, and therefore require higher thermal conductivity to transfer heat from the battery cells.

## Conclusions

This study, for the very first time, tackled the challenge of designing the battery housing of EVs using lattice structures, considering multidisciplinary requirements. A multi-objective TO-based framework is proposed that utilises surface-based TPMS lattices to identify solutions with competing mechanical and thermal requirements. It bridges the research gap by embedding thermal convection into the TO framework, whilst accounting for thermal conduction and mechanical stiffness simultaneously. Consequently, it enables the optimisation of EV battery housing based on the TPMS lattice with high heat convection capacity. The effectiveness of the proposed framework has been demonstrated through the generation of a Pareto front. The solution offering the best mechanical performance was $1.7 \times$ better than that of the benchmark case (i.e. uniform or ungraded lattice). The optimised designs outlined the importance of the elliptical material distribution for improving the mechanical stiffness under torsional loading. Supported by various fundamental theories, the results presented shed light into the mechanisms involved in the strategic material reallocation, which are valuable to design iterations. Through this effort, the benefits of a mathematically rigorous optimisation framework are evident and the widespread adoption of these would tremendously benefit the engineering community. Both designers and engineers are encouraged to draw inferences from the insights provided herein to enhance multidisciplinary structures with similar end-use requirements. This work introduces the concept of applying lattice structures to advance the battery systems in EVs and more widely within other battery-powered transport. It is going to have a significant impact, especially with the emergency of batteries possessing ultra-high energy density, as required for advanced applications like electric aircraft. The utilisation of TPMS lattice facilitates the support-free additive manufacturing of the design and offers unique opportunities to adopt such processes within the EV industry. Moreover, our framework can be readily extended to other physics, including diverse constraints and employing various lattice topologies to enable highly efficient engineering solutions.

## Data Availability

The datasets used and analysed during the current study are available from the corresponding author on reasonable request.

## References

[1] Government sets out path to zero emission vehicles by 2035 — gov.uk (accessed 06 December 2023). https://www.gov.uk/government/news/government-sets-out-path-to-zero-emission-vehicles-by-2035.

[2] EU ban on sale of new petrol and diesel cars from 2035 explained | News | European Parliament — europarl.europa.eu (accessed 06 Decmber 2023). https://www.europarl.europa.eu/news/en/headlines/economy/20221019STO44572/eu-ban-on-sale-of-new-petrol-and-diesel-cars-from-2035-explained.

[3] Jia, C. *et al.* Health-conscious deep reinforcement learning energy management for fuel cell buses integrating environmental and look-ahead road information. *Energy* **290**, 130146. 10.1016/j.energy.2023.130146 (2024).

[4] Berdichevsky, G., Kelty, K., Straubel, J. & Toomre, E. The tesla roadster battery system: Tesla motors. http://large.stanford.edu/publications/coal/references/docs/tesla.pdf (2007).

[5] Kang, S., Kim, J., Jang, Y. & Lee, K. Welding deformation analysis, using an inherent strain method for friction stir welded electric vehicle aluminum battery housing, considering productivity. *Appl. Sci.*10.3390/app9183848 (2019).

[6] Heinzen, T., Plaumann, B. & Kaatz, M. Influences on vibration load testing levels for BEV automotive battery packs. *Vehicles* **5**, 446–463. 10.3390/vehicles5020025 (2023).

[7] Shui, L. *et al.* Design optimization of battery pack enclosure for electric vehicle. *Struct. Multidiscip. Optim.* **58**, 331–347. 10.1007/s00158-018-1901-y (2018).

[8] Zhang, J., Kang, H., Wu, K., Li, J. & Wang, Y. The impact of enclosure and boundary conditions with a wedge-shaped path and air cooling for battery thermal management in electric vehicles. *Int. J. Energy Res.* **42**, 4054–4069. 10.1002/er.4122 (2018).

[9] Li, W. *et al.* Intelligent optimization methodology of battery pack for electric vehicles: A multidisciplinary perspective. *Int. J. Energy Res.* **44**, 9686–9706. 10.1002/er.5600 (2020).

[10] Dhoke, A. & Dalavi, A. A critical review on lightweight design of battery pack enclosure for electric vehicles. *Int. J. Sustain. Transp. Technol.* **4**, 53–62. 10.31427/ijstt.2021.4.2.2 (2021).

[11] Yeong, W. Y., Sing, S. L., Aman, B. Feasibility study on topological optimisation and additive manufacturing of an electric vehicle battery housing. *Mapp. Intim*. 10.21203/rs.3.rs-914347/v1 (2021).

[12] Li, J. & Zhang, H. Thermal characteristics of power battery module with composite phase change material and external liquid cooling. *Int. J. Heat Mass Transf.* **156**, 119820. 10.1016/j.ijheatmasstransfer.2020.119820 (2020).

[13] Schmerler, R. *et al.* Multifunctional FRP-aluminum foam production setup for battery housings of electric vehicles. *Technol. Lightweight Struct. (TLS)* **4**, 9–17. 10.21935/tls.v4i1.115 (2020).

[14] Plocher, J. & Panesar, A. Review on design and structural optimisation in additive manufacturing: Towards next-generation lightweight structures. *Mater. Des.* **183**, 108164. 10.1016/j.matdes.2019.108164 (2019).

[15] Pan, C., Han, Y. & Lu, J. Design and optimization of lattice structures: A review. *Appl. Sci.* **10**, 6374. 10.3390/app10186374 (2020).

[16] Wang, J. & Panesar, A. Machine learning based lattice generation method derived from topology optimisation. *Addit. Manuf.* **60**, 103238. 10.1016/j.addma.2022.103238 (2022).

[17] Yeranee, K. & Rao, Y. A review of recent investigations on flow and heat transfer enhancement in cooling channels embedded with triply periodic minimal surfaces (tpms). *Energies*10.3390/en15238994 (2022).

[18] Yan, C., Hao, L., Hussein, A. & Raymont, D. Evaluations of cellular lattice structures manufactured using selective laser melting. *Int. J. Mach. Tools Manuf.* **62**, 32–38. 10.1016/j.ijmachtools.2012.06.002 (2012).

[19] Yang, L. *et al.* Mechanical response of a triply periodic minimal surface cellular structures manufactured by selective laser melting. *Int. J. Mech. Sci.* **148**, 149–157. 10.1016/j.ijmecsci.2018.08.039 (2018).

[20] Panesar, A., Abdi, M., Hickman, D. & Ashcroft, I. Strategies for functionally graded lattice structures derived using topology optimisation for additive manufacturing. *Addit. Manuf.* **19**, 81–94. 10.1016/j.addma.2017.11.008 (2018).

[21] Wu, J., Sigmund, O. & Groen, J. P. Topology optimization of multi-scale structures: A review. *Struct. Multidiscip. Optim.* **63**, 1455–1480. 10.1007/s00158-021-02881-8 (2021).

[22] Wang, C. *et al.* Concurrent design of hierarchical structures with three-dimensional parameterized lattice microstructures for additive manufacturing. *Struct. Multidiscip. Optim.* **61**, 869–894. 10.1007/s00158-019-02408-2 (2020).

[23] Zhang, Y., Xiao, M., Li, H., Gao, L. & Chu, S. Multiscale concurrent topology optimization for cellular structures with multiple microstructures based on ordered simp interpolation. *Comput. Mater. Sci.* **155**, 74–91. 10.1016/j.commatsci.2018.08.030 (2018).

[24] Ali, M. A. & Shimoda, M. Toward multiphysics multiscale concurrent topology optimization for lightweight structures with high heat conductivity and high stiffness using matlab. *Struct. Multidiscip. Optim.*10.1007/s00158-022-03291-0 (2022).

[25] He, L. *et al.* Battery pack, vehicle and energy storage device US Patent. https://patents.google.com/patent/US11183729B2/en. (2021).

[26] COMSOL Multiphysics Version 6.1. https://www.comsol.com. (2023).

[27] COMSOL Documentation. https://doc.comsol.com/5.5/doc/com.comsol.help.sme/sme_ug_solid.07.65.html.

[28] MATLAB (R2022b). https://www.mathworks.com. (2022).

[29] Laloui, L. & Rotta Loria, A. F. Chapter 3 - heat and mass transfers in the context of energy geostructures. In *Analysis and Design of Energy Geostructures* (eds Laloui, L. & Rotta Loria, A. F.) 69–135 (Academic Press, New York, 2020). 10.1016/B978-0-12-816223-1.00003-5.

[30] Rao, Z. & Wang, S. A review of power battery thermal energy management. *Renew. Sustain. Energy Rev.* **15**, 4554–4571. 10.1016/j.rser.2011.07.096 (2011).

[31] Dong, G., Tang, Y. & Zhao, Y. F. A 149 line homogenization code for three-dimensional cellular materials written in matlab. *J. Eng. Mater. Technol.*10.1115/1.4040555 (2018).

[32] Marler, R. T. & Arora, J. S. Survey of multi-objective optimization methods for engineering. *Struct. Multidiscip. Optim.* **26**, 369–395. 10.1007/s00158-003-0368-6 (2004).

[33] Chang, C.-T. Multi-choice goal programming with utility functions. *Eur. J. Oper. Res.* **215**, 439–445. 10.1016/j.ejor.2011.06.041 (2011).

[34] Bendsøe, M. P. & Sigmund, O. *Topology Optimization: Theory, Methods, and Applications* (Springer Science & Business Media, 2019).

